# Woody Species Diversity, Composition, and Regeneration Status of Abbo Sacred Forest, Southern Ethiopia

**DOI:** 10.1155/2022/9112578

**Published:** 2022-11-04

**Authors:** Alemayehu Yigeremu, Mesfin Woldearegay

**Affiliations:** Department of Biology, College of Natural and Computational Sciences, Debre Birhan University, P. O. Box 445, Debre Berhan, Ethiopia

## Abstract

This study was conducted in Abbo Sacred Forest in Wonsho district, Sidama Zone of Southern Nations, Nationalities, and People's Region (SNNPR), southern Ethiopia, to investigate the woody species diversity, vegetation structure, and regeneration status of the forest. A systematic sampling design was employed to collect vegetation data. Sixty quadrats of 20 m × 20 m were laid at every 200 m interval between each quadrat and 400 m apart between each line transect following the altitudinal gradient. Quadrats of 20 m × 20 m were used to record DBH and the height of all woody plant species reaching a height of ≥2 m and a DBH of ≥2 cm. For the inventory of seedlings and saplings, five subplots of 2 m × 2 m, one at the center and the other four on each corner of the main quadrat, were used. DBH, height, seedling, and sapling density of woody species were recorded in each quadrat. The data were analyzed by using Shannon–Wiener diversity and equitability indices, and the structural analysis was carried out based on frequency, density, DBH, height, and basal area/ha^−1^. The importance value index was also computed. Regeneration status was computed by comparing the density data of saplings and seedlings with those of mature trees. A total of 63 woody species belonging to 56 genera and 35 families were identified. Three endemic species to Ethiopia were recorded. Analysis of selected woody species showed diverse population structures. The findings of this study revealed that small trees and shrubs dominated the forest, suggesting its status under a secondary stage of development. Some woody species require urgent conservation measures. Therefore, local and regional stakeholders should integrate and work together to develop and implement sound conservation and management strategies that encourage the sustainable utilization of forest resources.

## 1. Introduction

Ethiopia has a unique ecological setting with considerably varied soil, climate, and biological resources. The elevation ranges from the highest peak to 4,533 m a.s.l. down to 125 m below sea level. The topographic settings of the country stretch over a high altitudinal range covering mountains, flat-topped plateaus, deep gorges, and valleys as well as aquatic and wetland environments [1]. Owing to this great geographical diversity and climatic variations, the country has been endowed with diversified fauna and flora that make it an important regional center of biological diversity and endemism [1]. The flora of Ethiopia is very heterogeneous and consists of 5757 species and subspecies of vascular plants, of which 633 are endemic [2]. The forest resources of the country are declining from time to time. However, recently by adopting a new definition of forest, the forest area covers 15.7% of the country's total land area [3] but high forest areas still do not cover a significant proportion and the rate of deforestation is 92,000 ha per year [3]. The biodiversity and ecosystems of the country are highly threatened by multiple factors such as increased demand for resources and products, agricultural expansion, climate change, and the introduction and expansion of invasive alien species [1, 4].

Sacred forests are the remnant surviving forest patches that preserve indigenous and old-growth forest trees with cultural and/or religious importance to the local people [5, 6]. Sacred forests are found in many countries all over the world and are particularly common in parts of Asia and Africa [7]. Sacred forests are playing a significant role in biodiversity conservation and combating climate change [8] since exploitation is relatively prohibited due to spiritual and cultural taboos and rules and customs. Local communities in different parts of the world lived in harmony with nature and conserved valuable biodiversity around them [9]. This is mainly because their environment is closely related to their survival and development and hence they do focus on protection and sustainable biodiversity conservation [10]. Indigenous communities usually have well-developed local knowledge and skills in conservation and sustainable utilization of local biodiversity [9].

Sacred forests represent an important tradition of conserving specific land areas that have cultural and religious importance [11]. These forests contain many rare plant species and store high carbon than non-sacred habitats [8]. Moreover, sacred forests or traditional protected areas are found to have higher tree cover, higher plant species diversity, and greater biomass than non-sacred sites [12]. In some African countries like Tanzania, sacred forests have greater woody species richness and taxonomic diversity than state-managed forest reserves. In Ghana, sacred forests are sanctuaries for rare, threatened, or endangered species, and in the central region of the Republic of Benin, sacred forests have higher tree species diversity than in non-sacred areas [7]. Ethiopia has many sacred forests that are protected by indigenous communities. They are commonly found in southwest, central, and northern Ethiopia where they have been protected by strong local beliefs and norms as sacred sites and church forests [13].

Sacred forests can have significant contributions to local communities by providing material, non-material, and regulating services. Many scholars advocate the importance of these forests for biodiversity conservation and global ecological services such as carbon sequestration [14, 15]. Despite the importance of sacred forests for global biodiversity conservation and local-level benefits, many sacred natural sites have continued to be overlooked by governments, conservation institutions, and broader civil society. Therefore, many sacred forests still have no legal protection and face threats from agricultural expansion and other land-use changes associated with population growth and economic development [6, 16]. Understanding the floristic diversity, vegetation structure, and natural regeneration status of the species in sacred forests helps to obtain basic information for designing and implementing sound management and conservation strategies for sustainable utilization of the resource. To this end, the Abbo Sacred Forest in southern Ethiopia has never been scientifically studied for its plant diversity, structural analysis, and regeneration status and thus is targeted for this investigation. The overall objective of this study was to investigate the floristic composition, species diversity, and regeneration status of woody species in Abbo Sacred Forest.

## 2. Materials and Methods

### 2.1. Description of the Study Area

**Figure 1:**
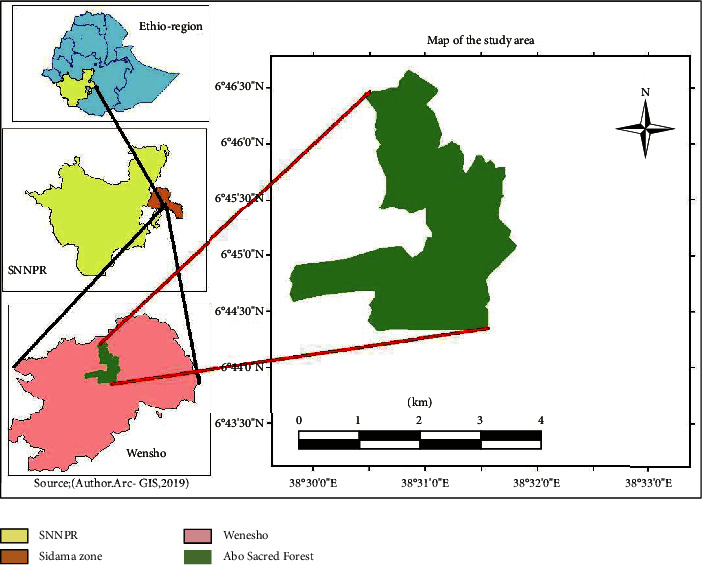
Location of the study area.

The study was carried out in Abbo Sacred Forest, Wonsho district, Sidama Zone of Southern Nations, Nationalities, and People's Region (SNNPR), southern Ethiopia. The study area is found 59 km southeast of Hawassa town, the capital of SNNPR. It is situated between 6° 14′ to 7° 18′ N latitude and 39° 12′ to 39° 14′ *E* longitude (Figure 1). The topography of the forest is characterized by an undulating mountainous landscape and extends over an altitudinal range from 1700 to 3200 m above sea level. The study area is characterized by bimodal rainfall distribution where the short rain occurs between March and May while the long rain starts at the end of June and extends up to October [17]. The dry periods extend from December to February. The mean annual rainfall of the study area was 1130 mm while the mean annual temperature was 17°C [17].

Wonsho district is divided into two major agro-climatic zones, namely, mid-highland (locally called Woyna Dega) (75%) and highland (locally called Dega) (25%). The district has a total population of 104,474 (53,058 males and 51,416 females) [18]. The people living in the district have different ethnic compositions, among others, Sidama, Oromo, Amhara, Gurage, Tigrie, and Wolayita. Sidama constitutes the dominant ethnic group [18]. This indicates the existence of active interaction and exchange of cultural practices and indigenous knowledge among the people.

The district covers a total land area of about 14,640 ha where 231.53 ha is covered with natural forest. The natural vegetation of the study area is moist evergreen Afromontane forest, which is characterized by closed strata of evergreen trees. This moist evergreen Afromontane forest occurs between 1500 and 2600 m, with annual rainfall between 700 and 2000 mm [1, 19]. The tree canopies usually contain a mixture of *Pouteria adolfi-friederici* (Engl.) Baehni and other broad-leaved species. Other major canopy trees in the study area include *Syzygium guineense* (Willd.) DC subsp. *afromontanum* F. White, *Vepris dainellii* (Pic.Serm.) Kokwaro, *Prunus africana* (Hook. f.) Kalkm., *Psydrax schimperiana* (A. Rich.) Bridson, *Polyscias fulva* (Hiern) Harms, *Olea capensis* L., *Macaranga capensis* (Baill.) Sim, and *Ocotea kenyensis* (Chiov.) Robyns and Wilczek. The total area of Abbo Sacred Forest is 92.33 ha [20].

### 2.2. Sampling Design and Data Collection

A reconnaissance survey was made from September 20 to 30, 2019, at the study site to obtain a general impression of the physiognomy of the forest and identify sampling sites. A systematic sampling design was employed for vegetation and environmental data collection [21, 22]. The sampling sites were distributed along eight-line transects laid following the altitudinal gradient. The line transects were laid in different directions and the number of quadrats in each line was 7, 7, 9, 8, 6, 10, 6, and 7, respectively. Once the first quadrat was established randomly, subsequent independent quadrats were laid along line transects at every 200 m interval between each quadrat and 400 m apart between each line transect. The plots were placed 50 m away from the forest margin to avoid edge effects related to disturbance. A total of 60 quadrats were established each having 20 × 20 m (400 m^2^) dimension for an inventory of trees and shrubs, and five 2 × 2 m (4 m^2^) subquadrats, one at the center and the other four on each corner of the main quadrat, were laid for an inventory of saplings and seedlings. Seedling and sapling data of woody plants were collected to determine the regeneration status of the forest.

In each quadrat, the height and diameter at breast height (DBH) of all woody species with a height of ≥2 m and diameter at breast height ≥2 cm were measured and recorded. If a tree or shrub branches at breast height or below, the diameter of each branch was measured separately and the average was taken. The height of all woody plants was measured using calibrated stick marked at 0.5 m intervals and 4 m long and visually estimated, and the diameter at breast height was measured using diameter tape. Woody plant species with DBH <2 cm and a height of 1–2 m were considered saplings while those woody plants with a height of <1 m were considered seedlings [23]. Altitude and geographic coordinates of each quadrat were measured using Garmin 60 GPS. Growth forms (tree, shrub, and liana) of plants were recorded and voucher specimens were collected, dried, pressed, and identified. Identification of specimens was carried out both in the field and later at the National Herbarium (ETH) Addis Ababa University using taxonomic keys in the Flora of Ethiopia and Eritrea and also by comparison with authenticated specimens. The nomenclature of the plant specimens followed the Flora of Ethiopia and Eritrea [24–30].

### 2.3. Data Analysis

#### 2.3.1. Diversity Indices

Biological diversity can be measured using different indices. The Shannon–Wiener diversity index is the most widely used measure of species diversity [21, 31] because it combines species richness with species evenness (relative abundance). The Shannon diversity index (H′) was calculated using the following formula:

(1) $$\begin{array}{c} H^{\prime }=-\overset{s}{\underset{i=1}{\sum }}Pi\ln Pi, \end{array}$$

where H′ = Shannon diversity index, *s* = the number of species, Pi = the proportion of individuals of the *i*^th^ species expressed as a proportion of total cover in the sample, and ln = the natural logarithm. The Shannon evenness index (*J*) was also calculated using the following formula:

(2) $$\begin{array}{c} J=\frac{H^{\prime }}{H^{\prime }max}=\;\frac{H^{\prime }}{\ln s}, \end{array}$$

where *J* = Shannon equitability or evenness index, H′ = Shannon–Wiener diversity index, H′ max = the maximum level of diversity possible within a given population, which equals In *s*, *s* = the number of species, and In = the natural logarithm.

#### 2.3.2. Structural Analysis

Structural analysis of the forest was carried out based on frequency, density, DBH, height, and basal area per hectare as described in [21, 22]. Both DBH and height were classified into seven DBH and height classes, and the density distribution of woody species in each class was computed to describe the population structure of selected woody species in the forest [31]. The basal area (BA) of trees was computed by BA = (d/200)^2^*π*, where BA = basal area in m^2^ per hectare, *d* = diameter at breast height in cm, and *π* = 3.14. The importance value index (IVI) of each tree species was computed as indicated in [22] based on the formula: IVI = relative density (RD) + relative frequency (RF) + relative dominance (RDO) where

(3) $$\begin{array}{c} \text{RD}=\left(\frac{\text{Number of individuals of a tree species}}{\text{Total number of all tree species}}\right)\times 100,\\ \text{RF}=\left(\frac{\text{Frequency of a tree species}}{\text{Total frequency of all tree species}}\right)\times 100,\\ \text{RDO}=\left(\frac{\text{Dominance of a tree species}}{\text{Dominance of all tree species}}\right)\times 100. \end{array}$$

Density and frequency were computed as follows [32]:

(4) $$\begin{array}{c} \text{Density}=\left(\frac{\text{Total no}.\text{ of individuals of a species found}}{\text{Total area sampled}}\right),\\ \text{Frequency}=\left(\frac{\text{No}.\text{ of quadrats in which a species is found}}{\text{Total no}.\text{ of quadrats sampled}}\right)\times 100. \end{array}$$

#### 2.3.3. Regeneration Status

The regeneration status of Abbo Sacred Forest was computed by comparing the density data of seedlings and saplings with those of matured trees following the techniques described in [33, 34]. It was categorized as “good” regeneration if seedlings > saplings > mature trees; “fair” regeneration, if mature trees > saplings > seedlings; “poor” regeneration, if the species survives only in the sapling stage (saplings less than, more than, or equal to mature trees) with no seedlings; and “no” regeneration if the species is absent in both seedling and sapling stage but present as a mature tree.

## 3. Results and Discussion

### 3.1. Floristic Composition and Species Diversity

Sixty-three woody plant species belonging to 56 genera and 35 families were recorded from Abbo Sacred Forest (Table 1). Out of these, 37 (58.7%) species were trees followed by 20 (31.8%) shrubs and 6 (9.5%) lianas. Fabaceae were considered the most dominant family represented by five species (7.9%), followed by Euphorbiaceae and Rubiaceae with four species each (6.4%), Asteraceae, Lamiaceae, Moraceae, Oleaceae, and Rutaceae with three species each (4.8%), and eight families with two species each (3.2%), while the remaining 19 families had a single species representation. The findings of this study showed that Abbo Sacred Forest has higher woody species richness than Wabero forest (47 species) [35] and Ades forest (48 species) [36] but its species richness was lower than that of Sirso forest (74 species) [37] and Wurg forest (76 species) [38] in Ethiopia. Variations in species composition among different forests could be attributed to topographic variables such as elevation, latitude, and longitude [39]. Moreover, regeneration success and competition are also important factors that shape the species composition of forests. Fabaceae were considered the dominant family in Abbo Sacred Forest which could be related to its efficient and successful dispersal mechanisms and better adaptation to a wide range of ecological conditions [28]. Fabaceae were also found to be dominant in other Afromontane forests in Ethiopia [36, 40, 41]. Species such as *Maytenus addat* (Loes.) Sebsebe, *Vepris dainellii* (Pichi-Serm.) Kokwaro, and *Millettia ferruginea* (Hochst.) Bak. are endemic to Ethiopia. These endemic plant species accounted for 4.8% of the total species recorded. The overall Shannon–Wiener diversity and evenness values of Abbo Sacred Forest were 2.99 and 0.75, respectively, which indicated the high species diversity of the forest. This result was higher than that of Yemrehane Kristos Church Forest (H' = 2.88) [40] and Munessa natural forest (H' = 2.66) [41]. The differences in species diversity of the forests could be attributed to the variation in altitude, disturbance history, and other environmental factors of the study areas [38]. On the other hand, the reduction of species diversity could be an indication of the increased disturbance caused by domestic animals and human intervention at maturity or the early stage of regeneration. According to Kent and Coker [31], the Shannon–Wiener diversity index normally varies between 1.5 and 3.5 and rarely exceeds 4.5. Thus, the H′ value of Abbo Sacred Forest resides within the normal range.

### 3.2. Tree and Shrub Density

The total density of trees and shrubs with DBH >2 cm was 1319.17 individuals ha^−1^ in Abbo Sacred Forest. As indicated in Table 2, ten most abundant woody species accounted for 76.5% of the total density. The density of trees and shrubs with DBH >10 cm was 517.17 ha^−1^ while that of species with DBH >20 cm was 256 ha^−1^. The ratio of trees and shrubs with DBH >10 cm to DBH >20 cm was 2.02. Abbo Sacred Forest showed relatively high woody species density compared to some other Afromontane forests in Ethiopia such as Menfeskidus Monastery forest (395.8 individuals ha^−1^) [42], Yemrehane Kristos Church Forest (506.6 individuals ha^−1^) [40], and Kafta Sheraro National Park (466 individuals ha-1) [43] while its density was lower than that of Wurg forest (1745.27 individuals ha^−1^) [38] and Agama forest (1446 individuals ha^−1^) [44]. This could be due to differences in landscape topographic gradients and habitat qualities related to ecological requirements of component tree species in the respective forests. As well as selective removal of tree species for various purposes such as charcoal production, house construction and expansion of agricultural land. Heavy grazing/browsing of domestic animals and harvesting of woody plants at an early growth stage for various purposes by the local people might cause a reduction in plant species diversity over time [45]. The ratio of the density of trees and shrubs with DBH >10 cm to DBH >20 cm is taken as a measure of size class distribution. The value of this ratio was 2.02 in Abbo Sacred Forest which is higher than that of some other Afromontane forests in Ethiopia like Agama forest (1.98) [44], Kenech forest (1.8) [33], and Majang forest (1.4) [46], indicating a predominance of small-sized individuals that start to grow following excessive cuttings or other anthropogenic disturbances.

### 3.3. Diameter at Breast Height and Height Class Distribution

**Figure 2:**
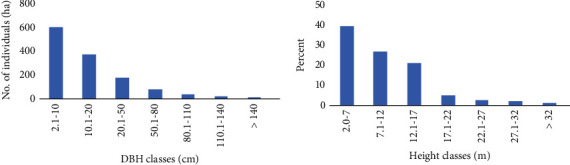
DBH and height class distribution of woody species in Abbo Sacred Forest.

The height and DBH class distribution of all individuals in the different size classes indicated an inverted J-shaped pattern (Figure 2). DBH and height class distribution showed an inverted J-shaped distribution which is a general pattern of normal population structure where the majority of the species had a larger number of individuals at lower DBH and height classes and gradually decreased in number towards the higher DBH and height classes. Such inverted J-shaped distributions across DBH and height classes are indicative of a stable population structure of the forest [4, 33]. A similar pattern of population structure was reported in previous studies from different Afromontane forests of Ethiopia [37, 38, 42, 44, 47].

### 3.4. Frequency

*Croton macrostachyus* Del. was the most frequently occurring species with 70.5% of all quadrats sampled followed by *Brucea antidysentrica* J.F. Mill. (68%), *Maytenus arbutifolia* (A. Rich.) Wilczek. (45.9%), *Syzygium guineense* subsp. *afromontanum* (Willd.) DC. (43.4%), *Vernonia auriculifera* Hiern. (33.6%), and *Ritchiea albersi* Gilg. (26.2%). The least frequent species in the forest include *Ficus thonningi* Blume., *Ocimum lamifolium* Hochst. ex Benth., *Rhamus prinoides L* ′Herit., *Clematis simensis* Fresen., *Dodenaea angustifolia* L.f., and *Calpurnia aurea* (Alt.) Benth., each constituting 0.8%. Frequency provides an approximate indication of the homogeneity and heterogeneity of a stand [21]. This study shows a higher number of species in the lower frequency classes and a lower number of species in the higher frequency classes suggesting the presence of floristic heterogeneity in the forest. Similar results were also reported by other studies in Ethiopia [33, 44, 46]. On the other hand, a small number of species in lower frequency classes and a large number of species in higher frequency classes show floristic homogeneity or similar species composition.

### 3.5. Basal Area

The basal area of a stand of trees is the sum of the cross-sectional surface areas of each tree measured at breast height (1.3 m above ground surface). It is used to explain the crowdedness of a stand of trees [45]. The total basal area of Abbo Sacred Forest was 67.85 m^2^·ha^−1^. This basal area was higher than that reported by [48] for Bonga (47 m^2^·ha^−1^), Harena (49 m^2^·ha^−1^), Berhane-Kontir (54 m^2^·ha^−1^), and [42] for Menfeskidus Monastery (56.30 m^2^·ha^−1^) Afromontane forests of Ethiopia but it was much lower than that reported for Wurg (126.47 m^2^·ha^−1^) [38] and Agama (80.08 m^2^·ha^−1^) [44] Afromontane forests of Ethiopia. This variation could be related to the differences in the conservation efforts, exposure to deforestation, climatic conditions, and geographical location of the forests. Moreover, the high basal area of Abbo Sacred Forest could be related to the presence of very few, large-sized individuals in the canopy tree. About 40% of the total basal area was contributed by *Syzygium guineense* ssp. *afromontanum* (Willd.) DC. (16.47 m^2^·ha^−1^, 24.27%), and *Pouteria adolfi-friederici* (Engl.) Baehni (10.46 m^2^·ha^−1^, 15.41%). Tree species with the largest contribution in the basal area can be considered the most important species, and hence these two species were found to be important in Abbo Sacred Forest.

### 3.6. Importance Value Index (IVI)

The importance value index (IVI) indicates the relative ecological significance of a tree species in a stand and also used for setting the priority/rank of species for management and conservation practices [46, 49]. It reflects the degree of dominance and abundance of a given species compared to other species in the study area [33]. IVI combines data from relative density, relative frequency, and relative dominance [21], and hence it is considered the most realistic aspect of vegetation study by many ecologists [32]. The results of this study showed higher IVI values for *Syzygium guineense* ssp. *afromontanum* (Willd.) DC. and *Croton macrostachyus* Del. than for any other species in the forest (Table 3). Therefore, in this study, the IVI results also confirmed that the aforementioned species are the most important/dominant species in the study area.

### 3.7. Population Structure

**Figure 3:**
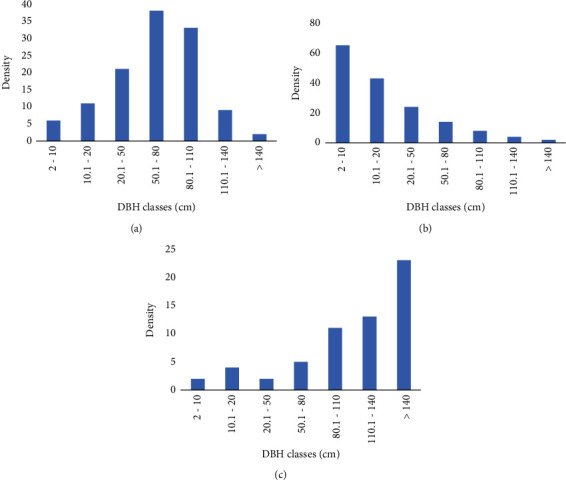
(a–c) Representative patterns of species population structure in Abbo Sacred Forest.

Population structure analysis of woody species in Abbo Sacred Forest resulted in three major representative patterns of the density distribution of trees across different DBH classes. The patterns of species population structure provide useful information about the population dynamics and regeneration status of species in a forest to design appropriate management and conservation strategies [33]. The first pattern was a bell-shaped distribution formed by species with a fewer number of individuals in the lower and higher DBH classes and a larger number of individuals in the middle classes (Figure 3(a)). This pattern of distribution was represented by *Croton macrostachyus* Del.*, Ekebergia capensis* Sparrm.*, Erythrina abyssinica* Lam. ex DC., and *Pittosporum viridiflorum* Sims. This distribution follows a Gaussian type of distribution pattern, where the species had a low density of individuals in the first and second DBH classes and a gradual increase in the number of individuals in the middle classes followed by a subsequent decline in the higher DBH classes. This pattern of distribution shows a poor reproduction and recruitment of species and also a decline in the number of large-sized trees. Selective cutting of large-sized individuals for various purposes, mainly for timber, construction, farm implements, and firewood, could be the reason for the decline in the number of large-sized trees. Similar results were reported from previous studies in the Afromontane forests of Ethiopia [38, 44, 47].

The second pattern was an inverted J-shaped distribution formed by species with a larger number of individuals in the lower DBH classes, followed by a gradual decrease towards the higher DBH classes (Figure 3(b)). This pattern of distribution was represented by *Syzygium guineense* ssp. *afromontanum* (Willd.) DC.*, Psydrax schimperiana* (A. Rich.) Bridson*, Ocotea kenyensis* (Chiov.) Robyns and Wilczek., and *Macaranga capensis* (Baill.) Sim. This type of distribution pattern is an indicator of stable and healthier population dynamics of the species in the forest [46, 47, 49]. It is characteristic of species with high potential for reproduction and shade-tolerant trees that maintain a more or less constant rate of recruitment and seem to be a self-maintaining plant population.

The third pattern was a J-shaped distribution formed by species with a lower number of individuals in the lower DBH classes, followed by a gradual increase towards the higher DBH classes (Figure 3(c)). This pattern of distribution was represented by *Pouteria adolfi-friederici* (Engl.) Baehni*, Polyscias fulva* (Hiern) Harms.*, Olea capensis* L., and *Prunus africana* (Hook. f.) Kalkm. These species show poor reproduction and hampered regeneration status that could be attributed to the fact that either most trees are not producing seeds because of age or there are losses due to predators after reproduction [48]. Similar findings were reported by previous studies in the Afromontane forests of Ethiopia [38, 44]. In general, the overall representative population structures of species in Abbo Sacred Forest are indicators of the ultimate need for appropriate conservation activities to improve the overall regeneration status of all species in the forest. However, the first priority should be given to those species with no seedlings and saplings.

## 4. Conclusion

Abbo Sacred Forest has a relatively rich diversity of woody species (63 species) when compared to some other Afromontane forests of Ethiopia. The overall diversity and evenness values of the forest were 2.99 and 0.75, respectively. Of all the species recorded in the forest, three species, namely, *Maytenus addat* (Loes.) Sebsebe*, Vepris dainellii* (Pichi-Serm.) Kokwaro, and *Millettia ferruginea* (Hochst.) Bak., are endemic to Ethiopia that constituted 4.76% of the total species recorded in the forest. Structural analysis and regeneration status assessment of woody species indicated an overall healthy ecological condition of the forest. However, a detailed analysis of individual species revealed that some species showed abnormal population structure and poor reproduction and hampered regeneration status which needs careful conservation and management activities by the respective stakeholders to maintain the healthy condition of the forest. In addition, these forests are the remnant forest patches that contain mature trees which could serve as seed sources. Therefore, the role of these forests to rehabilitate/restore the nearby degraded areas as well as the ethnobotanical roles of the plant species could be recommended for future studies.

## Figures and Tables

**Table 1:** List of woody plant species recorded from Abbo Sacred Forest.

Species name	Family	Local name^*∗*^	Habit^*∗∗*^
*Achyranthes aspera* L.	Amaranthaceae	Nolle	H
*Acokanthera schimperi* (A. DC.) Schweinf.	Apocynaceae	Kararcho	T
*Albizia schimperiana* Oliv.	Fabaceae	Maticho	T
*Berasama abyssinica* Fresen.	Melianthaceae	Teberako	T
*Borassus aethiopum* Mart.	Arecaceae	Satcho	T
*Brucea antidysenterica* J.F. Mill.	Simaroubaceae	Hatabicho	T
*Calpurnia aurea* (Alt.) Benth.	Fabaceae	Ceketa	S
*Canthium lucidum* R. Br.	Rubiaceae	Werarcho	S
*Casimiroa edulis* La Liave	Rutaceae	Kazmera	T
*Celtis africana* Burm. f.	Ulmaceae	Shilicho	T
*Clematis longicauda* Steud. ex A. Rich	Ranunculaceae	Unknown	L
*Clematis simensis* Fresen.	Ranunculaceae	Unknown	L
*Clutia abyssinica* Jaub. and Spach.	Euphorbiaceae	Binijile	S
*Commiphora schimperi* (Berg) Engl.	Burseraceae	Gatancho	S
*Cordia africana* Lam.	Boraginaceae	Wadcho	T
*Croton macrostachyus* Del.	Euphorbiaceae	Mesincho	T
*Dodonea angustifolia* L. f.	Sapindaceae	Etancho	S
*Dovyalis abyssinica* (A. Rich.) Warb.	Flacourtiaceae	Kuke	S
*Ehretia cymosa* Thonn.	Boraginaceae	Gidincho	T
*Ekebergia capensis* Sparrm.	Meliaceae	Garbicho	T
*Embelia schimperi* Vatke.	Myrsinaceae	Qanqo	L
*Erythrina abyssinica* Lam. ex DC.	Fabaceae	Welako	T
*Euphorbia abyssinica* Gmel.	Euphorbiaceae	Cercho	T
*Fagaropsis angolensis* (Engl.) Dale.	Rutaceae	Godicho	T
*Ficus sur* Forssk.	Moraceae	Qilitto	T
*Ficus thonningi* Blume.	Moraceae	Dinbecho	S
*Ficus vasta* Forssk.	Moraceae	Qilitto	T
*Galiniera saxifraga* (Hochst.) Bridson	Rubiaceae	Danshicho	T
*Grewia ferruginea* Hochst. ex A. Rich.	Tiliaceae	Fokonch	T
*Grewia villosa* Willd.	Tiliaceae	Qiximalcho	S
*Hagenia abyssinica* (Brace) J.F. Gmel.	Rosaceae	Dadako	T
*Ilex mitis* (L.) Radlk.	Aquifoliaceae	Meqicho	T
*Jasminum grandiflom* L.	Oleaceae	Unknown	L
*Justicia schimperiana*	Acanthaceae	Cikicho	S
*Lactuca inermis* Forssk.	Asteraceae	Hadhesa	H
*Landolphia buchananii* (Hall.f.) Stapf.	Apocynaceae	Unknown	L
*Leucas stachydiformis* (Hochst. ex Benth.) Briq.	Lamiaceae	Shemella	S
*Macaranga capensis* (Baill.) Sim.	Euphorbiaceae	Felako	T
*Maesa lanceolata* Forssk.	Myrsinaceae	Gowacho	T
*Maytenus addat* (Loes.) Sebsebe	Celastraceae	Buiacuco	S
*Maytenus arbutifolia* (A. Rich.) Wilczek.	Celastraceae	Cuco	S
*Millettia ferruginea* (Hochst.) Bak.	Fabaceae	Hengedicho	T
*Ocimum lamiifolium* Hochst. ex Benth.	Lamiaceae	Damakesie	S
*Ocotea kenyensis* (Chiov.) Robyns and Wilczek.	Lauraceae	Shoecho	T
*Olea capensis L*.	Oleaceae	Ejersa	T
*Olea welwitschii* (Knobl.) Gilg and Schellenb.	Oleaceae	Setamo	T
*Pavetta oliveriana* Hiern.	Rubiaceae	Welincho	T
*Phoenix reclinata* Jacq.	Arecaceae	Saticho	T
*Phytolacca dodencandra L* ′Herit.	Phytolaccaceae	Haranjicho	L
*Pittosporum viridiflorum* Sims.	Pittosporaceae	Boncho	T
*Plectranthus igniarius* (Schweinf) Agnew.	Lamiaceae	Tontoncho	S
*Polyscias fulva* (Hiern) Harms.	Araliaceae	Kobrie	T
*Pouteria adolfi-friederici* (Engl.) Baehni	Sapotaceae	Dugucho	T
*Prunus africana* (Hook. f.) Kalkm.	Rosaceae	Dongicho	T
*Psydrax schimperiana* (A. Rich.) Bridson	Rubiaceae	Werarcho	T
*Rhamnus prinoides L* ′Herit.	Rhamnaceae	Tedcho	S
*Ritchiea albersii* Gilg.	Capparidaceae	Kokolicho	S
*Rumex abyssinicus* Jacq.	Polygonaceae	Shisho	H
*Rytigynia neglecta* (Hirn) Robyns	Rubiaceae	Unknown	S
*Senna didymobotrya (*Fresen.) Irwin and Barneby.	Fabaceae	Hameshohaka	S
*Solanum incanum L*.	Solanaceae	Borbodhecho	S
*Syzygium guineense* Subsp. *afromontanum* (Willd.) DC.	Myrtaceae	Duwancho	T
*Vepris dainellii* (Pichi-Serm.) Kokwaro	Rutaceae	Betrikcho	T
*Vernonia amygdalina* Del.	Asteraceae	Hacho	T
*Vernonia auriculifera* Hiern.	Asteraceae	Rejicho	S
*Vernonia myriantha Hook. f.*	Astraceae	Jejeko	S

^*∗*^Wolaytigna. ^*∗∗*^H = herb; T = tree; S = shrub; L = liana.

**Table 2:** Density (individuals ha^−1^) and percentage contribution of ten most abundant woody species in Abbo Sacred Forest.

Species name	Density	%
*Brucea antidysenterica* J.F. Mill.	196.67	14.91
*Calpurnia aurea* (Alt.) Benth.	60.00	4.55
*Croton macrostachyus* Del.	217.08	16.46
*Euphorbia abyssinica* Gmel.	43.75	3.32
*Leucas stachydiformis* (Hochst. ex Benth.) Briq.	52.08	3.95
*Maytenus addat* (Loes.) Sebsebe	53.33	4.04
*Maytenus arbutifolia* (A. Rich.) Wilczek.	167.92	12.73
*Ritchiea albersii* Gilg.	53.75	4.07
*Syzygium guineense* subsp. *afromontanum* (Willd.) DC.	61.25	4.64
*Vernonia auriculifera* Hiern.	103.33	7.83
Other 53 species	310	23.5
Total	1319.17	100

**Table 3:** Importance value index (IVI) of trees and shrubs in Abbo Sacred Forest.

Species	Relative density (RD)	Relative frequency (RF)	Relative dominance (RDO)	IVI
*Syzygium guineense* ssp. *afromontanum* (Willd.) DC.	4.62	5.46	24.27	34.35
*Croton macrostachyus* Del.	16.54	8.86	3.67	29.07
*Brucea antidysentrica* J.F. Mill.	14.97	8.45	2.53	25.95
*Maytenus arbutifolia* (A. Rich.) Wilczek.	12.78	6.77	2.74	22.30
*Pouteria adolfi-friederici* (Engl.) Baehni	0.64	2.26	15.41	18.31
*Vernonia auriculifera* Hiern.	7.21	4.52	0.50	12.23
*Ocotea kenyensis* (Chiov.) Robyns and Wilczek.	0.38	3.71	5.40	9.49
*Ritchiea albersi* Gilg.	4.00	4.29	0.78	9.07
*Leucas stachydiformis* (Hochst. ex Benth.) Briq.	3.96	4.58	0.49	9.03
*Calpurnia aurea* (Alt.) Benth.	4.57	2.85	0.47	7.89
*Maytenus addat* (Loes.) Sebsebe	4.06	2.34	1.45	7.85
*Ekebergia capensis* Sparrm.	0.72	2.31	4.54	7.57
*Bersama abyssinica* Fresen.	0.51	5.47	1.45	7.42
*Ephorbia abyssinica* Gmel.	3.45	2.68	0.70	6.83
*Albizia schimperiana* Oliv.	0.54	0.32	5.65	6.51
*Maesa lanceolata* Forssk.	2.60	3.13	0.70	6.43
*Milletia ferruginea* (Hochst.) Bak.	1.81	1.03	3.27	6.10
*Senna didymobotrya* (Fresen.) Irwin and Barneby.	3.16	2.58	0.24	5.98
*Olea capensis L*.	0.76	0.10	4.27	5.13
*Grewia ferruginea* Hochst. ex A. Rich.	2.70	1.93	0.27	4.89
*Hygenia abyssinica* (Brace) J.F. Gmel.	0.86	1.65	1.87	4.37
*Prunus africana* (Hook. f.) Kalkm.	0.51	1.34	1.90	3.74
*Itex mitis* (L.) Radlk.	0.60	0.51	2.05	3.16
Other species	8.06	22.86	15.38	46.30
Total	100.00	100.00	100.00	

## Data Availability

The data used to support the findings of this study are included within the article and are also available from the corresponding author upon request.
